# Evaluation of sample size effect on the identification of haplotype blocks

**DOI:** 10.1186/1471-2105-8-200

**Published:** 2007-06-14

**Authors:** Dai Osabe, Toshihito Tanahashi, Kyoko Nomura, Shuichi Shinohara, Naoto Nakamura, Toshikazu Yoshikawa, Hiroshi Shiota, Parvaneh Keshavarz, Yuka Yamaguchi, Kiyoshi Kunika, Maki Moritani, Hiroshi Inoue, Mitsuo Itakura

**Affiliations:** 1 Department of Bioinformatics, Division of Life Science Systems, Fujitsu Limited, 1-5-2, Higashishinbashi, Minato-ku, 105-7123, Tokyo, Japan; 2 Section for Diabetes, Genotyping Division, Genetic Diversification Analysis Project, Japan Biological Information Consortium (JBIC), Tokyo, Japan; 3 Division of Genetic Information, Institute for Genome Research, the University of Tokushima, 3-18-15, Kuramoto-cho, 770-8503, Tokushima, Japan; 4 Division of R&D Solution, Fujitsu Nagano Systems Engineering Limited, 380-3813, Nagano, Japan; 5 Department of Endocrinology and Metabolism, Kyoto Prefectural University of Medicine Graduate School of Medical Sciences, 465, Kajii-cho, Hirokoji-Kawaramachi, Kamigyo-ku, Kyoto, 602-8566, Japan; 6 Department of Ophthalmology and Visual Neuroscience, Institute for Health Biosciences, the University of Tokushima, 3-18-15, Kuramoto-cho, Tokushima, 770-8503, Japan

## Abstract

**Background:**

Genome-wide maps of linkage disequilibrium (LD) and haplotypes have been created for different populations. Substantial sharing of the boundaries and haplotypes among populations was observed, but haplotype variations have also been reported across populations. Conflicting observations on the extent and distribution of haplotypes require careful examination. The mechanisms that shape haplotypes have not been fully explored, although the effect of sample size has been implicated. We present a close examination of the effect of sample size on haplotype blocks using an original computational simulation.

**Results:**

A region spanning 19.31 Mb on chromosome 20q was genotyped for 1,147 SNPs in 725 Japanese subjects. One region of 445 kb exhibiting a single strong LD value (average |D'|; 0.94) was selected for the analysis of sample size effect on haplotype structure. Three different block definitions (recombination-based, LD-based, and diversity-based) were exploited to create simulations for block identification with *θ *value from real genotyping data. As a result, it was quite difficult to estimate a haplotype block for data with less than 200 samples. Attainment of a reliable haplotype structure with 50 samples was not possible, although the simulation was repeated 10,000 times.

**Conclusion:**

These analyses underscored the difficulties of estimating haplotype blocks. To acquire a reliable result, it would be necessary to increase sample size more than 725 and to repeat the simulation 3,000 times. Even in one genomic region showing a high LD value, the haplotype block might be fragile. We emphasize the importance of applying careful confidence measures when using the estimated haplotype structure in biomedical research.

## Background

There is a great interest in using genetic association studies to identify the disease-susceptibility variants related to the common complex diseases. To design these studies appropriately, it is important to understand the feature of linkage disequilibrium (LD) in candidate genes or genomic regions of interest [1]. Several studies have shown that the human genome contains regions of high LD value with low haplotype diversity by a small number of SNPs [2-4]. These regions are called haplotype block, each of which reflects the descent from a single ancient ancestral chromosome. The construction of a haplotype block is one way to reduce the complexity of the problem of association mapping of the common complex diseases.

Haplotype blocks are defined computationally by various algorithms. In general, they are classified into the following categories: recombination-based, LD-based, and diversity-based methods. These block definitions are consistent with a block-covered sequence, which is considered a block as a part of the genomic sequence. However, the haplotype block border is not usually stable, and blocks can fall into sub-blocks within the border [5].

The general properties of haplotype block construction in the human genome are not well understood. Thus, the International Haplotype Map (HapMap) project has genotyped a huge number of SNPs in samples from subjects of Caucasian, African, and Asian descent to better understand the human haplotype structure [6]. Several studies (including HapMap) have shown differences in LD and haplotype block patterns in populations and chromosomes. In addition, it was revealed that SNPs ascertainment, selection and spacing could explain the observed block length [5,7], and that SNPs density has a crucial influence on the length of method-defined blocks [8]. Despite the extensive empirical studies on haplotype blocks, there is no definitive answer as to how sample size impacts the assessment of block structure. For example, a study on chromosome 21 examined 20 independent subjects from diverse populations [2]. Even for a relatively large data set, it contained only 275 individual samples, leading to 400 independent chromosomes [4]. Thus, it was possible that the detected block structure was dependent on the small number of samples, and this seemed a preliminary finding. It remains unclear how many individuals are needed to acquire reliable features of haplotype block [5,9].

In this study, we developed a simulation with random re-sampling from real genotyping data in 725 Japanese and introduced an original measurement of *θ *value for the identification of haplotype block defined by three algorithms. With the original measurement, we focused on haplotype block structure, especially within a high LD region. We further assessed the robustness of haplotype blocks estimated under the different sample size conditions.

## Results

### Selection of the analyzed region

**Figure 1:**
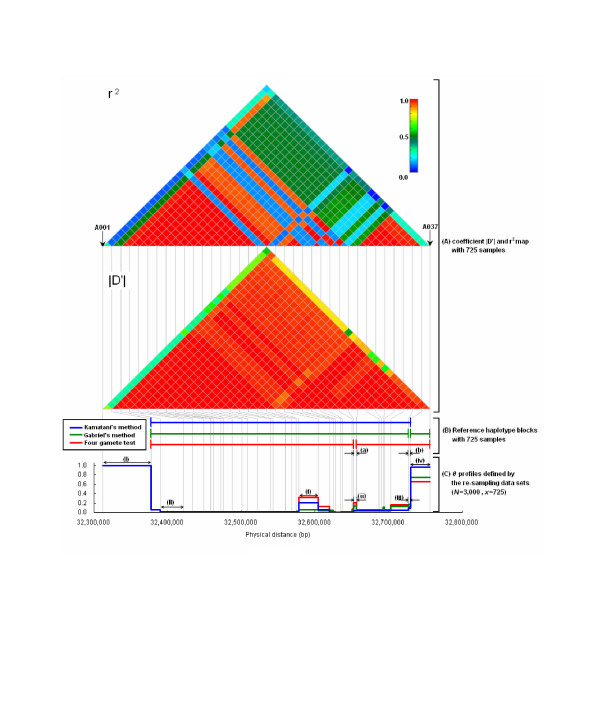
(A) Map of linkage disequilibrium (LD) coefficient |D'| (lower part) and r^2 ^(upper part) within the analyzed region on chromosome 20q11.22 (445 kb). A single strong |D'| block (average 0.94) is observed, but broken into small blocks with r^2^. (B) Structure of reference haplotype blocks with three different algorithms (Kamatani's method, Gabriel's method, and four-gamete test) using 725 samples. Each haplotype block is shown as blue (Kamatani's), green (Gabriel's), or red (four-gamete test) horizontal bars. Two gaps of (a) and (b) were observed in the analyzed region ((a) in the four-gamete test, and (b) in Gabriel's method). (C) *θ *profiles with the maximum number of samples (*x *= 725), and 3,000 repeats (*N *= 3,000). *θ *profiles show values between 0 and 1 based on its definition. The scale of *θ *profiles is shown on the vertical axis. The physical distance on chromosome 20q is shown on the horizontal axis. SNPs positions are presented as vertical gray bar through (A) to (C). Based on the complete identification of *θ *profiles, two SNPs intervals of (I) and (II) were selected for further analysis. We also selected four SNPs intervals from (i) to (iv), showing the variations of *θ *profiles, for further analysis. (A), (B), and (C) are illustrated at the same physical scale.

One region was selected on chromosome 20q11.22, in which a single strong |D'| block was observed without substantial recombination in the current population (Fig. 1A). However, the region was broken into small blocks by r^2^. The average values of |D'| and r^2 ^were 0.94 and 0.59, respectively. Based on NCBI human build 35, the total length was about 445 kb from 32,311,428 to 32,756,554 bp. This region was composed of 37 SNPs, and the average distance between SNPs was 12.4 kb. SNPs with MAF greater than 0.1 (average MAF 0.36) were used for subsequent study. Detailed information on selected SNPs is shown in Table 1.

**Table 1:** List and information of 37 SNPs in the analyzed region on chromosome 20q11.22

No.	dbSNP ID	Position	No. of used samples		Minor allele frequency			P value (HWE test)			P value (association test)		Odds ratio
							
			Control	Case	Control	Case	Overall	Control	Case	Overall	Allele	Genotype	
A001	rs819135	32,311,428	357	362	0.40	0.34	0.37	0.32	0.30	0.18	0.03	0.08	1.27
A002	rs6088466	32,377,195	358	361	0.46	0.50	0.48	0.84	0.79	0.99	0.17	0.37	1.16
A003	rs1205336	32,389,997	358	367	0.36	0.35	0.36	0.47	0.81	0.50	0.85	0.93	1.02
A004	rs3746455	32,420,877	357	367	0.36	0.35	0.35	0.72	0.87	0.72	0.86	0.98	1.02
A005	rs6058029	32,433,304	352	367	0.35	0.35	0.35	0.44	0.87	0.51	0.94	0.90	1.01
A006	rs6087579	32,448,816	354	367	0.36	0.35	0.36	0.78	0.87	0.76	0.86	0.98	1.02
A007	rs6579165	32,458,376	357	359	0.36	0.36	0.36	0.72	0.71	0.61	0.98	1.00	1.00
A008	rs4911420	32,462,315	358	365	0.36	0.35	0.35	0.80	0.84	0.75	0.74	0.95	1.04
A009	rs4277599	32,472,566	356	360	0.36	0.35	0.35	0.70	0.74	0.61	0.79	0.96	1.03
A010	rs2424992	32,475,721	354	366	0.36	0.35	0.35	0.67	0.80	0.63	0.85	0.97	1.02
A011	rs6120644	32,487,471	355	364	0.36	0.36	0.36	0.92	0.81	0.81	0.94	0.99	1.01
A012	rs3736762	32,500,997	357	361	0.36	0.36	0.36	0.72	0.87	0.71	0.96	0.99	1.01
A013	rs6059850	32,508,445	352	364	0.36	0.35	0.35	0.98	0.70	0.77	0.76	0.92	1.03
A014	rs6059856	32,521,615	355	367	0.36	0.35	0.36	0.92	0.87	0.85	0.85	0.98	1.02
A015	rs6059866	32,539,471	355	360	0.36	0.35	0.35	0.92	0.92	0.89	0.80	0.97	1.03
A016	rs6059868	32,543,121	357	366	0.36	0.35	0.35	0.72	0.91	0.75	0.85	0.97	1.02
A017	rs6088512	32,559,552	355	365	0.36	0.35	0.36	0.86	0.83	0.78	0.86	0.98	1.02
A018	rs6120669	32,568,689	356	367	0.36	0.35	0.36	0.88	0.87	0.83	0.83	0.98	1.02
A019	rs1122174	32,574,507	356	367	0.18	0.15	0.16	0.41	0.97	0.51	0.09	0.22	1.27
A020	rs6087592	32,578,164	353	367	0.35	0.36	0.35	0.66	0.96	0.79	0.87	0.92	1.02
A021	rs11167239	32,604,133	354	363	0.36	0.36	0.36	0.88	0.72	0.72	0.85	0.97	1.02
A022	rs6088527	32,619,502	354	361	0.36	0.35	0.36	0.90	0.48	0.56	0.83	0.90	1.02
A023	rs764597	32,624,886	357	365	0.36	0.36	0.36	0.89	0.65	0.67	0.92	0.97	1.01
A024	rs2889849	32,627,938	352	365	0.18	0.15	0.16	0.32	0.41	0.80	0.11	0.13	1.26
A025	rs932542	32,635,029	357	367	0.18	0.15	0.16	0.45	0.42	0.94	0.15	0.19	1.23
A026	rs2295444	32,637,544	354	360	0.36	0.35	0.36	0.93	0.47	0.65	0.73	0.80	1.04
A027	rs2378199	32,650,141	358	360	0.17	0.14	0.16	0.26	0.36	0.73	0.07	0.08	1.30
A028	rs6088536	32,652,767	358	359	0.36	0.36	0.36	0.97	0.65	0.77	0.88	0.93	1.02
A029	rs6141488	32,656,407	356	367	0.46	0.49	0.48	0.24	0.71	0.56	0.24	0.28	1.13
A030	rs6142210	32,686,673	355	361	0.46	0.49	0.48	0.26	0.56	0.70	0.28	0.27	1.12
A031	rs6088552	32,690,152	358	366	0.46	0.49	0.48	0.24	0.60	0.64	0.22	0.23	1.14
A032	rs7269596	32,692,724	352	365	0.47	0.50	0.48	0.27	0.64	0.65	0.26	0.29	1.13
A033	rs6087612	32,694,483	352	367	0.46	0.50	0.48	0.29	0.64	0.67	0.16	0.21	1.16
A034	rs4911158	32,703,173	357	363	0.46	0.50	0.48	0.31	0.56	0.74	0.18	0.22	1.15
A035	rs6087616	32,726,694	356	364	0.46	0.50	0.48	0.46	0.60	0.86	0.18	0.28	1.15
A036	rs1321306	32,730,040	353	365	0.46	0.49	0.48	0.22	0.71	0.54	0.23	0.26	1.13
A037	rs910870	32,756,554	355	360	0.25	0.23	0.24	0.56	0.64	0.93	0.31	0.45	1.13

Genome position is based on the NCBI build 35. The number of called sample is the number after excluding the undetermined sample(s) with TaqMan assay. HWE denotes Hardy-Weinberg equilibrium. 95% CI denotes 95% confidence interval. The crude odds ratio was calculated for the allele model.

### Use of a novel measurement: *θ *value

An original measurement (*θ *value) was used for block identification. The *θ *value represents the probability of whether a SNPs interval resides within or outside a haplotype block. The word "interval" as used here refers to the region between two adjacent SNPs (See Methods). Using the *θ *value with more than two block definitions allows an estimation of the suitable structure of the haplotype block in the region of interest.

**Figure 2:**
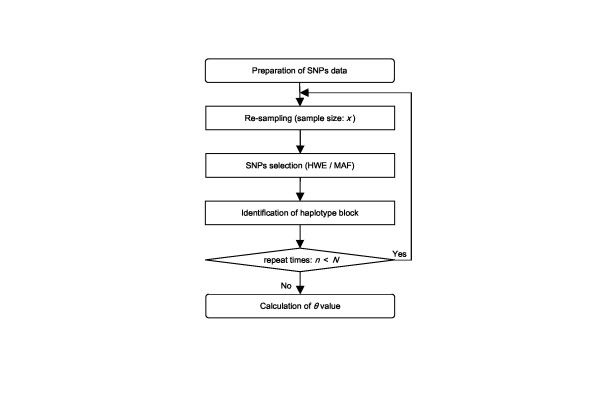
A flow chart of computational simulation. This flow chart outlines the basic algorithms including re-sampling, haplotype block definition, and block concordance (See Methods). *x *is the sample size in the re-sampling process. This parameter is increased from 50 to 700 in increments of 50, in addition to 10, 25, and 725. *n *is the number of repeat times of simulation under the maximum repeat time *N*. The *θ *value is an original measurement of the estimation of haplotype block, and is the ratio of times to *N *times when simulating the transition zone of block (See Materials and Methods). HWE denotes Hardy-Weinberg equilibrium, and MAF denotes minor allele frequencies.

In the analyzed region, different reference haplotype blocks were identified with three separate algorithms, despite a single strong |D'| value (Fig. 1B). To evaluate the discrepancy in block identification, *θ *profiles were calculated for all pairwise SNPs with a flow chart in Fig. 2.

The influence of the mixture of cases with control samples was examined first. *θ *profiles calculated with only controls (*x *= 358 and *N *= 3,000) were compared to the mixed data of controls and cases (*x *= 358 and *N *= 3,000). The mean square errors (MSE) between the two data sets was calculated, where MSE was the sum of the variance and squared bias of the estimates [10]. The observed MSE values were under 0.005 (0.5%) ranged from 0.00384 to 0.00305 (Additional file 1). Similarly, when *θ *profiles were compared with mixed data from 725 samples (*N *= 3,000), the MSE values were under 0.005, leading to a reasonable accuracy for the combination of the two groups (Additional file 1). This calculation suggested that the profound bias of the mixture of cases was rather low. In addition, there were no significant differences (Chi-square P < 0.01) in allele frequencies with all 37 SNPs in a case-control association study and all SNPs satisfied with HWE test (P < 0.05). As a result, the genotyping data from 367 cases and 358 controls were merged for a total of 725 samples of Japanese genotyping data.

With the maximum sample size of 725 subjects and 3,000 repeat times, *θ *profiles were plotted against the physical position of the SNPs (Fig. 1C). The complete concordance of haplotype blocks was observed in some intervals (e.g., (I) and (II)), but not in others (e.g., (i), (ii), (iii), and (iv)). It may be difficult to clearly identify the haplotype block even for the *θ *value. For subsequent analyses, two intervals, (I) and (II), were selected as the complete concordance regions of the block, and four intervals from (i) to (iv) were defined as incomplete concordance regions.

### Reference haplotype blocks and *θ *value

Although there was a difference among reference haplotype blocks across the analyzed region (Fig. 1B), *θ *profiles generated by the three algorithms were comparatively similar (Fig. 1C). We compared *θ *profiles with reference haplotype blocks defined by 725 samples.

In the interval (a), a reference haplotype block was disrupted by the four-gamete test, but it was continuous with the other two methods (Fig. 1B). This finding, supported by the intermediate r^2 ^value, identified interval (a) as the fragile region of haplotype block. In contrast, *θ *profiles showed low values (less than 0.2) with all three algorithms in the corresponding interval (interval (ii) in Fig. 1C). Namely, interval (a) was identified as the transition zone in 20% of 3,000 simulations of the *θ *values. Interpretation of the *θ *profiles reveals that interval (a) might be included in the transition zone of the block, and that LD might be disrupted against the estimation of Gabriel's and Kamatani's methods.

All reference haplotype blocks were continuous in the interval (i) (Fig. 1B). However, four-gamete test showed a *θ *value of 0.30 after 3,000 simulations and a divided interval (Fig. 1C). In addition, the *θ *value was not zero with the other two algorithms. Similar discrepancies were observed between the *θ *value and reference blocks defined by a single algorithm with two other intervals (iii) and (iv). It was quite difficult to clearly identify the block structure in these SNPs intervals. These results suggest that haplotype blocks might be fragile with one block algorithm, even if the region showed a single strong LD value defined by |D'|.

### Data simulation by *θ *value

**Figure 3:**
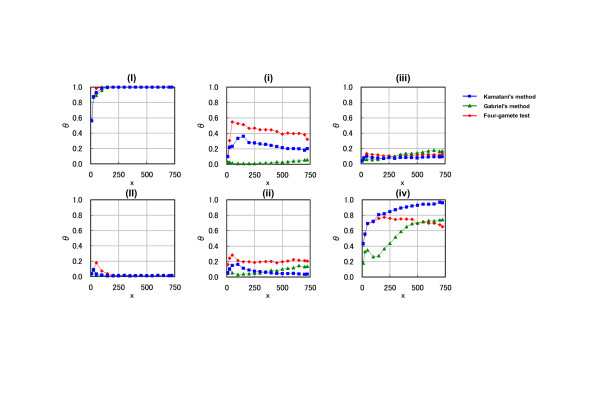
The effect of sample size on haplotype block characteristics under different block definitions: *θ *profiles versus sample number (*x*). The number of repeat times (*n*) was adjusted to 3,000 (*N *= 3,000). The labels of analyzed SNPs intervals are identical to those in Figure 1. Sample size (*x*) is shown on the horizontal axis. *θ *profiles (ratio of times to *N *times when simulating the transition zone of haplotype block) are shown on the vertical axis. The three labels represent different haplotype block definitions: blue square, Kamatani's method; green triangle, Gabriel's method; red diamond, four-gamete test.

To evaluate the effect of sample size on the identification of haplotype blocks, *θ *profiles were calculated with a variable number of sample sizes (*x*). Fig. 3 left side panels show the results obtained with intervals (I) and (II). Within these two intervals, complete concordance was observed between the *θ *value and reference block definitions. The *θ *value could increase or decrease to the maximum or minimum value (1 or 0) dependent on the sample size, and it converged when using more than 200 samples. This result suggests that sample size could have an effect on the identification of haplotype block, and it was not possible to obtain a reliable block identification for data with less than 200 samples.

There was also a difficulty with haplotype block definitions within the other four intervals ((i), (ii), (iii), and (iv)) in spite of the estimation by the *θ *value. The *θ *values in these intervals were more strongly influenced by sample size than those in intervals (I) and (II). In particular, the *θ *value did not converge even for calculations with more than 600 samples. This might imply that the precise identification of haplotype block was difficult for data with 725 samples. However, the exact number of samples required to reach the plateau value for precise evaluation of haplotype block remains unclear.

**Figure 4:**
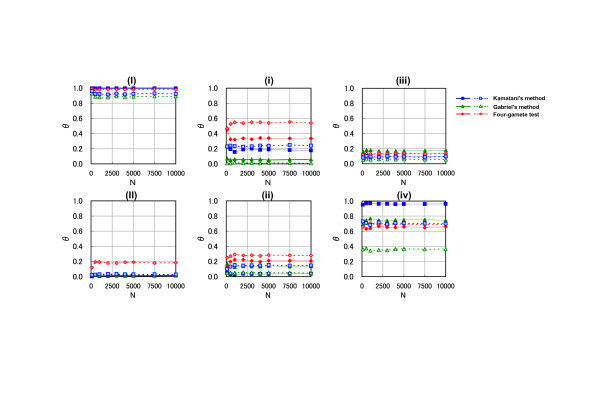
The effect of the number of simulation repeats on haplotype block characteristics under different block definitions: *θ *profiles versus the number of repeat times (*n*). The sample size (*x*) was adjusted by fixing it at 725 (solid lines) or 50 (dashed lines). The labels of analyzed SNPs intervals are identical to those in Figure 1. The number of repeat times (*n*) is shown on the horizontal axis. The simulation was repeated 10,000 times (*N *= 10,000) from 1, 100, 500, 1,000, 2,000, 3,000, 4,000, 5,000, and 7,500 times. *θ *profiles (the ratio of times to *N *times when simulating the transition zone of haplotype block) are shown on the vertical axis. The three labels represent different haplotype block definitions: blue square, Kamatani's method; green triangle, Gabriel's method; red diamond, four-gamete test.

*θ *profiles were also generated after changing the number of simulation times (*n*) up to the maximum repeat times of 10,000 (Fig. 4). The *θ *value converged and reached the plateau value in all algorithms when simulations were repeated 2,500 times. Thus, the *θ *value with 3,000 repeat times was reliable for block identification with the plateau value in the simulation. In the simulation of 50 samples (dashed lines), the *θ *value was not equal to the result in 725 samples (solid lines), even if the repeat times increased to the maximum (10,000). As a whole, these observations indicate that a sample size greater than 725 with a computational simulation of 3,000 times is required to obtain a converged *θ *value.

## Discussion

Given the importance of haplotype block for genome-wide association mapping, there is tremendous interest in constructing a haplotype block of the human genome. As a consequence, several questions have been raised about the nature of these blocks [3,4,6]. First, there are a number of variations in haplotype blocks based on the different block algorithms. In addition, several genetic factors, such as population, genetic marker density, and marker allele frequency, have contributed to the characteristics of haplotype blocks. However, it is not well known how many samples are sufficient to obtain reliable block characteristics [5,9,11]. To address this question, we genotyped 725 Japanese subjects with >1,000 SNPs on chromosome 20q. Additionally, we exploited a simple but refined simulation, which provided an original measurement (*θ *value) generated by random re-sampling from the real genotyping data.

The simulated data were derived from a single population, hence our study does not address all ethnic groups. However, we found that the number of samples had an effect on the characteristics of haplotype block, even in a limited region of the human chromosome in a single population. Indeed, the *θ *value could not reach plateau with less than 200 samples in some parts of the analyzed region. Recent works also indicated that sample size has a marked effect on the detection of observed haplotype blocks [5,11]. That study showed that a large sample size was required to minimize the bias in means based on |D'|, while trying to reduce the bias by bootstrapping [12]. To identify more general properties of haplotype block, a relatively larger number of samples would be required than those in this study. However, it is not feasible to increase the number of samples. Information on the variation of haplotype blocks is based on a trade-off between the accuracy of description, i.e., how much loss of information is acceptable, and the genotyping efforts needed to achieve this accuracy [5].

Another important and unsolved issue is the extent of block boundary conservation; namely, how fragile is the haplotype structure? To address this question, three different definitions of haplotype blocks were employed [13]. However, the primary concept of three algorithms was based on |D'| and focused on historical recombination, not allelic association. We could not avoid any bias resulting from a particular method. Although concordance of haplotype blocks exists in some regions, they are not common and might break up even if the region shows a single strong |D'| value [14]. As described above, the block did not have absolute boundaries and might be defined in different ways. In addition, our simulation provides only one of many possible approaches. It is difficult to compare the advantage of these algorithms because block identification was influenced by thresholds in the algorithms. It remains unclear how best to merge or integrate block definitions from different algorithms. We will not discuss the advantage of a particular algorithm as we view this as a separate problem.

One potential weakness is a possible ascertainment bias against SNPs allele frequency, which could influence haplotype block characteristics. SNPs with minor allele frequency greater than 10% were selected in this analysis, underscoring the variety of haplotype blocks. Therefore, our results cannot be used to infer the complete nature of haplotype blocks, because we referred to limited haplotype diversity from a bias in common SNPs.

## Conclusion

We developed a computational simulation that provides a realistic estimation of the observed genotyping data and attempted to unravel the underlying complexity of haplotype block. Based on this simulation, the sample size had an effect on the inferred haplotype block structure. For a valid description of haplotype blocks, further study will be required with a larger number of samples than presented here.

## Methods

### Preparation of genotyping data

Genomic DNA was collected from 725 Japanese subjects (male/female; 336/389), consisting of 358 healthy control subjects (male/female; 145/213) and 367 type 2 diabetic patients (male/female; 191/176). An examination of birthplace information established that all subjects were of full Japanese ancestry. Detailed clinical information of samples was previously described (Additional file 2) [15]. A total of 1,147 SNPs were selected in a 19.31 Mb region on chromosome 20q11.21-13.13 between D20S195 and D20S196. These SNPs were genotyped using a TaqMan SNP Genotyping Assay (Applied Biosystems, Foster City, CA) or a QuantiTect Probe PCR kit (Qiagen, Stanford, CA) according to the manufacturer's protocol. The fluorescence of VIC and FAM was measured with an ABI Prism 7900HT using Sequence Detector System (SDS) version 2.1 software (both from Applied Biosystems). The accuracy of genotyping was assessed by PCR-direct sequencing and showed 100% concordance as previously reported [15-17].

With the TaqMan method, genotype calls were identified by clustering the fluorescence intensity measurements for each SNP. All SNPs that were not members of genotyped clusters were eliminated. Expected genotyping accuracies were estimated with the quality score algorithm in SDS version 2.1. The intensity measurements were carefully checked by two independent researchers. In addition, the deviations of genotype distributions were evaluated by the Hardy-Weinberg equilibrium (HWE) test. This test was effective for identifying artifacts and improving data quality. Collectively, quality control was dependent on the reliability of the intensity measurements. The criteria used to judge reliability included calling rate, number of genotyped clusters, and consistency with the HWE test. Of the 1,147 SNPs genotyped, 103 were excluded and 1,044 passed the quality control criteria. An integrated system of Fujitsu Gene Discovery System (FGDS) version 2.0 (Fujitsu Ltd, Tokyo, Japan) was developed to deliver high-quality genotyping data [15-17].

### Linkage disequilibrium (LD) on chromosome 20q11.21-13.13

Linkage disequilibrium (LD) features along a 19.31 Mb contiguous segment on chromosome 20q11.21-13.13 were systematically characterized. FGDS version 2.0 was used to calculate the pairwise LD coefficients, |D'| and r^2 ^for the 1,044 SNPs that passed the quality control (Additional file 3).

The pairwise LD coefficient is the difference in haplotype frequency between the estimation of the expectation-maximization (EM) algorithm and calculation of the multiplication of allele frequencies. This coefficient is given as

D = x11 - p1q1,

where p1 and q1 are the frequencies of alleles A1 and B1 at loci A and B, and x11 is the frequency of haplotype A1B1. The standardized LD coefficient, r^2^, was calculated by the following formula [18]:

r^2 ^= D^2^/(p1p2q1q2),

where p2 and q2 are the frequencies of the other alleles at loci A and B. Lewontin's coefficient, |D'|, is described by the following formula:

|D'| = abs (D'), D' = D/D_max_,

where D_max _= minimum (p1q2, p2q1) when D is < 0, and where D_max _= minimum (p1q1, p2q2) when D is > 0 [19].

### Computational simulation

Figure 2 is a flow chart showing the procedure of computational simulation. The simulation consisted of re-sampling, haplotype block definition, and block concordance, including the process evaluating the effect of sample size (*x*).

#### Re-sampling

A re-sampling method was developed to replace the real genotyping data for assessment of the variation of haplotype blocks based on sample size. Sample size was randomly selected on the basis of SNP markers to permit the same sampling from the genotyping data from the 725 original samples. This re-sampling method is quite similar to the Mersenne Twister bootstrap method with random sampling for the generation of random numbers [20].

The conditions of HWE could change in the simulation with re-sampling, although the consistency of HWE existed in the genotyping data from the original 725 samples. SNPs with Chi-square P < 0.05 by HWE test were excluded from the simulation data to reduce the influence of HWE. Similarly, SNPs with minor allele frequencies (MAF) less than 0.10 were excluded from the simulation data.

#### Haplotype block definition

One purpose of the simulation was to evaluate the transition zone of the haplotype block. The transition zone of LD, in which continuous high LD values were disrupted by low LD values, was examined first [21].

Three different algorithms, recombination-based [9], LD-based [4], and diversity-based [22], were employed to define the haplotype block. The thresholds of three algorithms are shown in Table 2. Each algorithm provided different and complementary concepts. These three algorithms are described in detail in original papers and are briefly summarized below.

**Table 2:** Thresholds of three different algorithms to infer haplotype block

	Algorithms	Thresholds
1	Four-gametes test (recombination-based)	Threshold of forth gamete's haplotype frequency: 0.01
2	Gabriel's method (LD-based)	Confidence interval threshold for strong LD (lower side): 0.70 Confidence interval threshold for strong LD (upper side): 0.98 Upper confidence interval maximum for strong recombination: 0.90 Threshold of strong LD rate in region: 0.95
3	Kamatani's method (diversity-based)	Threshold of |D'| for initial clustering: 0.90 Threshold of haplotype frequency adding neighboring SNPs: 0.01

For the recombination-based method, a four-gamete test identifies a haplotype block as having the lowest frequency among the four phases of haplotypes consisting of pairwise SNPs [9]. The generation of genetic recombination in an interval of pairwise SNPs was indicated when the lowest frequency was greater than a threshold. Recombination was considered to have occurred if there were four haplotypes for any marker pair.

The LD-based method, Gabriel's method, is based on the LD coefficient |D'| and the 95% confidence interval (CI) [4]. Pairwise SNPs have a strong LD when the upper-side of 95% CI of |D'| is over 0.98, and the lower-side is over 0.70. In contrast, strong historical recombination exists when the upper-side of 95% CI of |D'| is less than 0.90. A haplotype block is defined as a region in which a small proportion (under 0.05) of SNPs shows strong historical recombination.

The foundations of the diversity-based Kamatani's method are the LD coefficient |D'| and haplotype frequency [22]. The haplotype block was constructed in two steps. First, an initial haplotype block was made consisting of all pairwise SNPs, in which |D'| was over 0.90. Using all SNPs in the initial block, the major haplotypes are identified by estimating the phase and frequency of haplotypes. In the second step, an adjacent SNP is added to the initial haplotype block, and the phase and frequency of haplotypes are estimated again. If another major haplotype is not recognized, an adjacent SNP is included in the initial haplotype block. The second step is repeated in the 5' and 3' directions until an additional major haplotype is generated. SNPHAP version 1.3.1 [23] was used to estimate haplotype phase and frequency.

#### Haplotype block concordance

The simulation procedure was repeated *N *times, with *N *ranging from 1 to 10,000. The incremental parameter of sampling times is represented as *n*. Original measurement for the identification of haplotype block, *θ *and *ρ*, were introduced to measure the correlation in haplotype structure between pairwise SNPs and provide a degree of confidence.

Let SNP_*j*,*k *_denote the interval between SNP_*j *_and SNP_*k*_. The number of times that each SNP_*j*,*k *_interval was included in or excluded from a reference haplotype block was calculated across all re-sampling samples. The reference haplotype block was defined using all 725 samples with each algorithm (Fig. 1B). The value obtained when SNP_*j*,*k *_was included in a transition zone of block (i. e., excluded from a reference block) was defined as *θ*_*j*,*k*_, the ratio of times to the total number of *N *times of the simulation. The relationship between *θ*_*j*,*k *_and *ρ*_*j*,*k *_was as follows:

θj,k=∑n=1Nρj,kn/N.
 MathType@MTEF@5@5@+=feaafiart1ev1aaatCvAUfKttLearuWrP9MDH5MBPbIqV92AaeXatLxBI9gBaebbnrfifHhDYfgasaacH8akY=wiFfYdH8Gipec8Eeeu0xXdbba9frFj0=OqFfea0dXdd9vqai=hGuQ8kuc9pgc9s8qqaq=dirpe0xb9q8qiLsFr0=vr0=vr0dc8meaabaqaciaacaGaaeqabaqabeGadaaakeaadaWcgaqaaGGaciab=H7aXnaaBaaaleaacqWGQbGAcqGGSaalcqWGRbWAaeqaaOGaeyypa0ZaaabCaeaacqWFbpGCdaqhaaWcbaGaemOAaOMaeiilaWIaem4AaSgabaGaemOBa4gaaaqaaiabd6gaUjabg2da9iabigdaXaqaaiabd6eaobqdcqGHris5aaGcbaGaemOta4KaeiOla4caaaaa@430A@

Let *ρ*_*j*,*k*_, which shows a value of 1 or 0, denote SNP_*j*,*k *_that is or is not included in the transition zone. When *ρ*_*j*,*k *_= 1, SNP_*j*,*k *_is within the transition zone (i.e., excluded from the reference haplotype block defined by 725 samples). When *ρ*_*j*,*k *_= 0, SNP_*j*,*k *_is outside the transition zone (i.e., included in the reference haplotype block defined by 725 samples). Let ρj,kn
 MathType@MTEF@5@5@+=feaafiart1ev1aaatCvAUfKttLearuWrP9MDH5MBPbIqV92AaeXatLxBI9gBaebbnrfifHhDYfgasaacH8akY=wiFfYdH8Gipec8Eeeu0xXdbba9frFj0=OqFfea0dXdd9vqai=hGuQ8kuc9pgc9s8qqaq=dirpe0xb9q8qiLsFr0=vr0=vr0dc8meaabaqaciaacaGaaeqabaqabeGadaaakeaaiiGacqWFbpGCdaqhaaWcbaGaemOAaOMaeiilaWIaem4AaSgabaGaemOBa4gaaaaa@33A1@ denote the *n*th data of *ρ*_*j*,*k *_in the total re-sampling number of *N *times. All profiles of *θ *were plotted as *θ*_*j*,*k *_against the physical position of SNPs sliding from *j *= A001 and *k = *A002 to *j *= A036 and *k = *A037.

In this analysis, all algorithms were implemented in Linux OS (Red Hat 9) using Perl. The simulation was run on an IA server with four 3.20 GHz Intel (R) Pentium (R) processors and 500 MB of RAM.

## Authors' contributions

DO developed the computational simulation method, and TT genotyped all DNA samples. DO and TT performed all statistical analyses. TT, DO and MI drafted the manuscript. NN, TY and HS cooperated the collection of DNA samples. All authors approved the final manuscript.

## Supplementary Material

Additional file 1 Supplementary Table 1 shows the mean square errors (MSE) in estimating the effect of the mixture of cases with controls. Click here for file

Additional file 2 Supplementary Table 2 shows the summary of 725 Japanese samples. Click here for file

Additional file 3 Supplementary Figure 1 shows the entire plot of linkage disequilibrium (LD) coefficient |D'| across chromosome 20q11.21-13.13 (19.31 Mb). Click here for file
