# Supplementary material for: Evaluation of sample size effect on the identification of haplotype blocks
Source: BMC Bioinformatics. 2007 Jun 14;8:200. doi: 10.1186/1471-2105-8-200 (PMC1913927; doi:10.1186/1471-2105-8-200)
Supplement: Additional file 1 — Supplementary Table 1 shows the mean square errors (MSE) in estimating the effect of the mixture of cases with controls. [file 1471-2105-8-200-S1.pdf]

Supplementary Table 1

Mean square error (MSE) in estimating the effect of the mixture of cases with controls

No.	SNPs interval	(A) 358 controls versus 358 mixed samples									(B) 358 controls versus 725 mixed samples								
		Four-gamate			Gabriel's			Kamatani's			Four-gamate			Gabriel's			Kamatani's		
		θ value		Square error	θ value		Square error	θ value		Square error	θ value		Square error	θ value		Square error	θ value		Square error
		358 controls	358 mixed		358 controls	358 mixed		358 controls	358 mixed		358 controls	725 mixed		358 controls	725 mixed		358 controls	725 mixed	
1	A001-A002	1.0000	1.0000	0.0000	1.0000	1.0000	0.0000	1.0000	1.0000	0.0000	1.0000	1.0000	0.0000	1.0000	1.0000	0.0000	1.0000	1.0000	0.0000
2	A002-A003	0.0677	0.0550	0.0002	0.0627	0.0543	0.0001	0.0580	0.0540	0.0000	0.0677	0.0537	0.0002	0.0627	0.0537	0.0001	0.0580	0.0533	0.0000
3	A003-A004	0.0790	0.0193	0.0036	0.0460	0.0163	0.0009	0.0093	0.0147	0.0000	0.0790	0.0140	0.0042	0.0460	0.0147	0.0010	0.0093	0.0137	0.0000
4	A004-A005	0.0123	0.0110	0.0000	0.0150	0.0103	0.0000	0.0050	0.0100	0.0000	0.0123	0.0117	0.0000	0.0150	0.0117	0.0000	0.0050	0.0113	0.0000
5	A005-A006	0.0073	0.0103	0.0000	0.0067	0.0100	0.0000	0.0047	0.0100	0.0000	0.0073	0.0117	0.0000	0.0067	0.0117	0.0000	0.0047	0.0113	0.0000
6	A006-A007	0.0067	0.0100	0.0000	0.0053	0.0097	0.0000	0.0043	0.0097	0.0000	0.0067	0.0107	0.0000	0.0053	0.0107	0.0000	0.0043	0.0103	0.0000
7	A007-A008	0.0067	0.0103	0.0000	0.0053	0.0097	0.0000	0.0043	0.0097	0.0000	0.0067	0.0107	0.0000	0.0053	0.0107	0.0000	0.0043	0.0103	0.0000
8	A008-A009	0.0067	0.0100	0.0000	0.0050	0.0093	0.0000	0.0043	0.0093	0.0000	0.0067	0.0107	0.0000	0.0050	0.0107	0.0000	0.0043	0.0103	0.0000
9	A009-A010	0.0063	0.0097	0.0000	0.0050	0.0090	0.0000	0.0043	0.0090	0.0000	0.0063	0.0107	0.0000	0.0050	0.0107	0.0000	0.0043	0.0103	0.0000
10	A010-A011	0.0063	0.0093	0.0000	0.0050	0.0087	0.0000	0.0043	0.0087	0.0000	0.0063	0.0107	0.0000	0.0050	0.0110	0.0000	0.0043	0.0103	0.0000
11	A011-A012	0.0043	0.0087	0.0000	0.0040	0.0080	0.0000	0.0037	0.0080	0.0000	0.0043	0.0107	0.0000	0.0040	0.0110	0.0000	0.0037	0.0103	0.0000
12	A012-A013	0.0057	0.0087	0.0000	0.0040	0.0080	0.0000	0.0037	0.0080	0.0000	0.0057	0.0110	0.0000	0.0040	0.0113	0.0001	0.0037	0.0107	0.0000
13	A013-A014	0.0037	0.0087	0.0000	0.0033	0.0080	0.0000	0.0033	0.0080	0.0000	0.0037	0.0100	0.0000	0.0033	0.0103	0.0000	0.0033	0.0097	0.0000
14	A014-A015	0.0037	0.0083	0.0000	0.0033	0.0077	0.0000	0.0033	0.0077	0.0000	0.0037	0.0087	0.0000	0.0033	0.0090	0.0000	0.0033	0.0083	0.0000
15	A015-A016	0.0037	0.0077	0.0000	0.0033	0.0073	0.0000	0.0033	0.0073	0.0000	0.0037	0.0077	0.0000	0.0033	0.0080	0.0000	0.0033	0.0073	0.0000
16	A016-A017	0.0043	0.0150	0.0001	0.0037	0.0073	0.0000	0.0033	0.0073	0.0000	0.0043	0.0087	0.0000	0.0037	0.0083	0.0000	0.0033	0.0073	0.0000
17	A017-A018	0.0040	0.0077	0.0000	0.0037	0.0073	0.0000	0.0033	0.0073	0.0000	0.0040	0.0083	0.0000	0.0037	0.0083	0.0000	0.0033	0.0073	0.0000
18	A018-A019	0.0047	0.0117	0.0000	0.0037	0.0073	0.0000	0.0033	0.0073	0.0000	0.0047	0.0097	0.0000	0.0037	0.0083	0.0000	0.0033	0.0073	0.0000
19	A019-A020	0.0163	0.0610	0.0020	0.0087	0.0090	0.0000	0.0117	0.0123	0.0000	0.0163	0.0237	0.0001	0.0087	0.0197	0.0001	0.0117	0.0120	0.0000
20	A020-A021	0.3083	0.3980	0.0080	0.0100	0.0177	0.0001	0.1097	0.2500	0.0197	0.3083	0.3237	0.0002	0.0100	0.0577	0.0023	0.1097	0.2017	0.0085
21	A021-A022	0.1467	0.1287	0.0003	0.0090	0.0133	0.0000	0.1313	0.0623	0.0048	0.1467	0.1210	0.0007	0.0090	0.0393	0.0009	0.1313	0.0573	0.0055
22	A022-A023	0.0083	0.0073	0.0000	0.0067	0.0103	0.0000	0.0063	0.0070	0.0000	0.0083	0.0077	0.0000	0.0067	0.0453	0.0015	0.0063	0.0050	0.0000
23	A023-A024	0.0183	0.0243	0.0000	0.0050	0.0057	0.0000	0.0063	0.0067	0.0000	0.0183	0.0093	0.0001	0.0050	0.0140	0.0001	0.0063	0.0040	0.0000
24	A024-A025	0.0050	0.0017	0.0000	0.0023	0.0023	0.0000	0.0020	0.0010	0.0000	0.0050	0.0023	0.0000	0.0023	0.0053	0.0000	0.0020	0.0013	0.0000
25	A025-A026	0.0070	0.0127	0.0000	0.0047	0.0023	0.0000	0.0033	0.0017	0.0000	0.0070	0.0103	0.0000	0.0047	0.0157	0.0001	0.0033	0.0013	0.0000
26	A026-A027	0.0087	0.0023	0.0000	0.0033	0.0007	0.0000	0.0030	0.0007	0.0000	0.0087	0.0040	0.0000	0.0033	0.0070	0.0000	0.0030	0.0013	0.0000
27	A027-A028	0.0427	0.0493	0.0000	0.0190	0.0180	0.0000	0.0163	0.0087	0.0001	0.0427	0.0700	0.0007	0.0190	0.0630	0.0019	0.0163	0.0087	0.0001
28	A028-A029	0.2800	0.1803	0.0099	0.1503	0.0640	0.0075	0.1857	0.0550	0.0171	0.2800	0.2070	0.0053	0.1503	0.1367	0.0002	0.1857	0.0357	0.0225
29	A029-A030	0.1377	0.0510	0.0075	0.1297	0.0567	0.0053	0.1143	0.0340	0.0065	0.1377	0.0500	0.0077	0.1297	0.0583	0.0051	0.1143	0.0340	0.0065
30	A030-A031	0.1307	0.0467	0.0071	0.1223	0.0390	0.0069	0.1120	0.0340	0.0061	0.1307	0.0550	0.0057	0.1223	0.0610	0.0038	0.1120	0.0340	0.0061
31	A031-A032	0.1393	0.0480	0.0083	0.1263	0.0380	0.0078	0.1120	0.0343	0.0060	0.1393	0.0543	0.0072	0.1263	0.0567	0.0049	0.1120	0.0347	0.0060
32	A032-A033	0.1990	0.0660	0.0177	0.1567	0.0590	0.0095	0.1177	0.0373	0.0065	0.1990	0.0870	0.0125	0.1567	0.1153	0.0017	0.1177	0.0360	0.0067
33	A033-A034	0.1260	0.0440	0.0067	0.1577	0.0583	0.0099	0.1143	0.0377	0.0059	0.1260	0.0463	0.0063	0.1577	0.0597	0.0096	0.1143	0.0370	0.0060
34	A034-A035	0.2500	0.1193	0.0171	0.1800	0.0633	0.0136	0.1410	0.0493	0.0084	0.2500	0.1563	0.0088	0.1800	0.1320	0.0023	0.1410	0.0423	0.0097
35	A035-A036	0.3080	0.1040	0.0416	0.3170	0.1153	0.0407	0.2427	0.0780	0.0271	0.3080	0.1313	0.0312	0.3170	0.1687	0.0220	0.2427	0.0927	0.0225
36	A036-A037	0.6677	0.7563	0.0079	0.6693	0.5843	0.0072	0.7240	0.8857	0.0261	0.6677	0.6523	0.0002	0.6693	0.7430	0.0054	0.7240	0.9620	0.0566
MSE				0.00384			0.00305			0.00374			0.00255			0.00176			0.00436

(A) θ profiles were calculated with 358 controls (N = 3,000) and compared with 358 mixed data of controls and cases (N = 3,000).

(B) θ profiles were calculated with 358 controls (N = 3,000) and compared with 752 mixed data of controls and cases (N = 3,000).
