# Supplementary material for: Evaluation of sample size effect on the identification of haplotype blocks
Source: BMC Bioinformatics. 2007 Jun 14;8:200. doi: 10.1186/1471-2105-8-200 (PMC1913927; doi:10.1186/1471-2105-8-200)
Supplement: Additional file 2 — Supplementary Table 2 shows the summary of 725 Japanese samples. [file 1471-2105-8-200-S2.pdf]

Supplementary Table 2

Summary of 725 Japanese samples

Phenotype	Normal controls	Type 2 diabetic patients
Number of samples	358	367
Male / Female	145 / 213	191 / 176
Age (years)	38.1 ± 14.2	64.1 ± 10.1
BMI (kg/m^2)	21.9 ± 2.9	23.6 ± 3.4
HbA1c (%)	4.7 ± 0.3	7.4 ± 1.4

Age, BMI, and HbA1c are represented as the means ± SD. BMI denotes body mass index.
