# Supplementary material for: Evaluation of sample size effect on the identification of haplotype blocks
Source: BMC Bioinformatics. 2007 Jun 14;8:200. doi: 10.1186/1471-2105-8-200 (PMC1913927; doi:10.1186/1471-2105-8-200)
Supplement: Additional file 3 — Supplementary Figure 1 shows the entire plot of linkage disequilibrium (LD) coefficient |D'| across chromosome 20q11.21-13.13 (19.31 Mb). [file 1471-2105-8-200-S3.pdf]

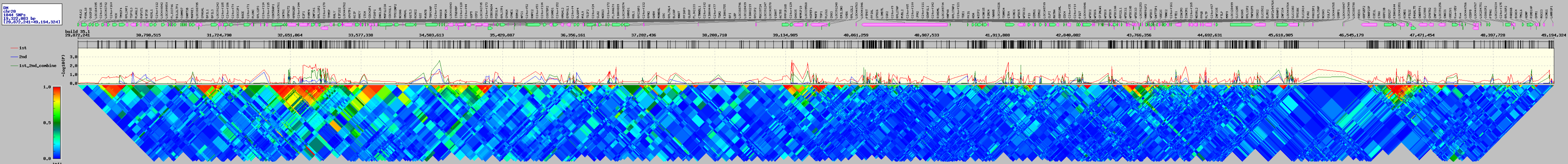
DM
chr20
1044 SNPs
19,322,083 bp
[29,872,241-49,194,324]
build 35.1
29,872,241
30,798,515
31,724,790
32,651,064
33,577,338
34,503,613
35,429,887
36,356,161
37,282,436
38,208,710
39,134,985
40,061,259
40,987,533
41,913,808
42,840,082
43,766,356
44,692,631
45,618,905
46,545,179
47,471,454
48,397,728
49,194,324
1st
2nd
1st_2nd_combine
-log10(P)
3.0
2.0
1.0
0.0
1.0
0.5
0.0
|D'|
